# Erythrocytes membrane fluidity changes induced by adenylyl cyclase cascade activation: study using fluorescence recovery after photobleaching

**DOI:** 10.1007/s00249-024-01707-x

**Published:** 2024-04-16

**Authors:** A. N. Semenov, A. E. Lugovtsov, S. A. Rodionov, Eu. G. Maksimov, A. V. Priezzhev, E. A. Shirshin

**Affiliations:** 1 Dynamics of Fluids, Department of Experimental Physics, Saarland University, Campus E2 6, 66123 Saarbrücken, Germany; 2 Faculty of Physics, M.V. Lomonosov Moscow State University, 1-2 Leninskie Gory, 119991 Moscow, Russia; 3 N.N. Priorov National Medical Research Center for Traumatology and Orthopedics, Priorova St. 10, 127299 Moscow, Russia; 4 World-Class Research Center “Digital Biodesign and Personalized Healthcare”, Sechenov First Moscow State Medical University, 8-2 Trubetskaya Str., 119991 Moscow, Russia; 5 Institute of Spectroscopy of the Russian Academy of Sciences, 5 Fizicheskaya Str., 108840 Moscow, Russia; 6 Faculty of Biology, M.V. Lomonosov Moscow State University, 1-12 Leninskie Gory, 119991 Moscow, Russia

**Keywords:** Erythrocytes membrane, Lipids diffusion, Adenylyl cyclase activation, FRAP

## Abstract

In this study, fluorescence recovery after photobleaching (FRAP) experiments were performed on RBC labeled by lipophilic fluorescent dye CM-DiI to evaluate the role of adenylyl cyclase cascade activation in changes of lateral diffusion of erythrocytes membrane lipids. Stimulation of adrenergic receptors with epinephrine (adrenaline) or metaproterenol led to the significant acceleration of the FRAP recovery, thus indicating an elevated membrane fluidity. The effect of the stimulation of protein kinase A with membrane-permeable analog of cAMP followed the same trend but was less significant. The observed effects are assumed to be driven by increased mobility of phospholipids resulting from the weakened interaction between the intermembrane proteins and RBC cytoskeleton due to activation of adenylyl cyclase signaling cascade.

**Graphical abstract:**

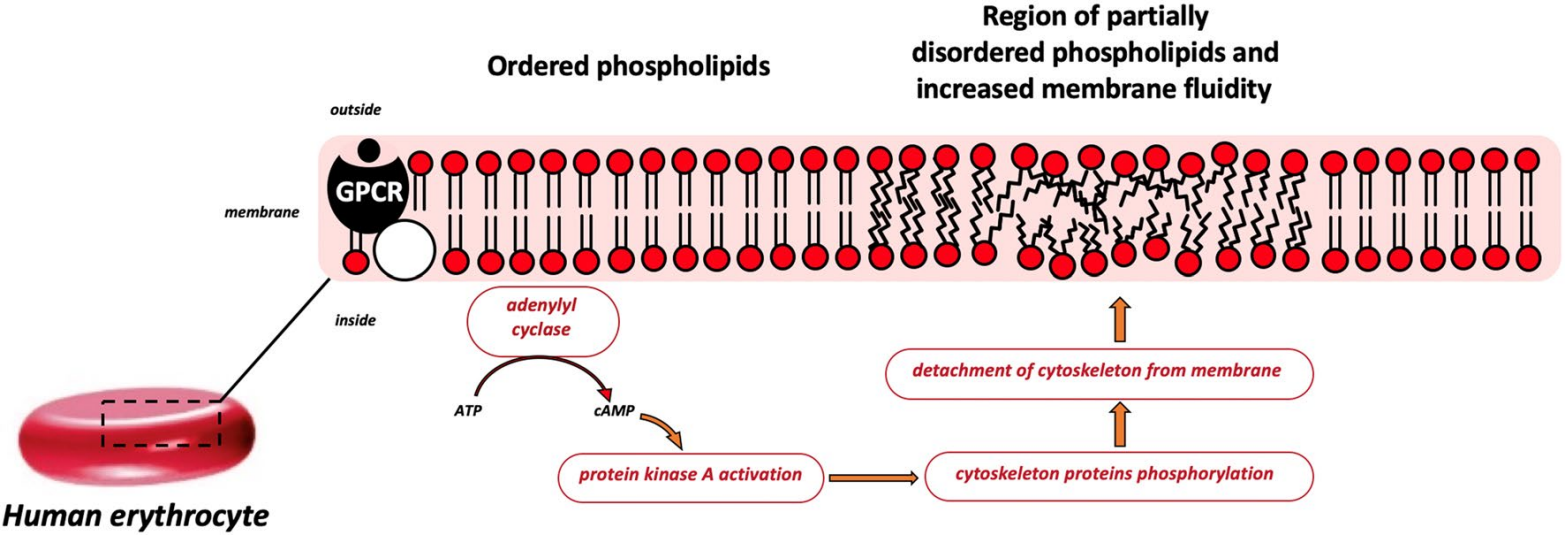

**Supplementary Information:**

The online version contains supplementary material available at 10.1007/s00249-024-01707-x.

## Introduction

Blood microcirculation requires the erythrocytes (red blood cells, RBCs) to be able to effectively change their shape while passing through capillaries with diameter less than RBC linear dimensions (Huisjes et al. 2018). In areas of the blood flow with high velocities and shearing rates, erythrocytes need to maintain integrity and preserve biconcave disk shape to provide optimal parameters of the blood flow (Mohandas et al. 1980). Such unique biomechanical feature is based on the deformability (Evans and La Celle 1975; Huisjes et al. 2018).

Recently, considerable experimental evidence has been accumulated about the mechanisms of adaptive regulation of RBC deformability in response to hypoxia, oxidative stress and various physiological requirements of the organism (Brun et al. 2022; Cilek et al. 2023). Majority of these mechanisms involve molecular signaling cascades, which are directed to the re-arrangements of the cytoskeleton proteins to weaken their interaction with each other and provide a better flexibility of the whole cytoskeletal network (Barshtein et al. 2023). Signaling can be triggered by the recognition of molecules by specific membrane receptors (Minetti et al. 2004; Mohandas and Gallagher 2008). External mechanical stress can also serve as a trigger for intracellular signalling transduction (Kuck et al. 2020; Simmonds et al. 2014) by inducing changes in the properties of membrane which mediates the activity of mechano-sensitive cation ion channel Piezo1 affecting RBC biomechanics (Kuck et al. 2022) or by modulation of the membrane potential via the Gardos effect (Jansen et al. 2021). The latter supports the context, in which changes in the state of the membrane can be recognized as a trigger within intracellular signaling. Hemoglobin oxygenation/deoxygenation status can serve as a signal for regulating the RBC volume and mechanical properties (Cilek et al. 2023; Uyuklu et al. 2009). Reactive oxygen species (ROS) (Diederich et al. 2018), gasotransmitters [nitric oxide (NO) and hydrogen sulfide (H2S)] (Muravyov et al. 2022), as well as local variations in the concentration of ATP and ADP in the bloodstream (Minetti et al. 2013) can activate the signaling cascades in erythrocytes despite their specific recognition is absent.

With all the variety of signaling agents, the intracellular signal transduction after the activation of the cascade is characterized by an increase in the catalytic activity of enzymes, which induce the conformational changes of the protein complexes. Regarding the molecular pathways responsible for RBC volume changes and the cytoskeletal re-organization, protein phosphorylation is considered to be a ubiquitous key mechanism (Cilek et al. 2023; Maneva and Maneva-Radicheva 2012). Residues of the proteins comprising joint connections between the actin/spectrin cytoskeleton and integral membrane proteins (namely, triad complexes ‘Band3-band4.2-ankyrin’ and ‘GlycophorinC-band4.1-p55’) are subjected to the phosphorylation by the set of kinases, which are activated upon signaling initiation (Manno et al. 1995). The corresponding protein complexes are disintegrated and the joints of cytoskeletal network are separated from the membrane. The latter results in the detachment of the cytoskeleton from the membrane leading to the temporary increase in RBC deformability. Such molecular mechanism of RBC deformability modulation is realized in the metabolic cascades involving protein kinase C, cAMP‐protein kinase A, cGMP‐nitric oxide, RhoGTPase, and MAP/ERK pathways (Cilek et al. 2023).

Previously it was demonstrated in our work (Semenov et al. 2019) and in works of other groups (Muravyov et al. 2011; Oonishi et al. 1997; Ramdani et al. 2015; Ugurel et al. 2019), that activation of various aspects of adenylyl cyclase cascade affects RBC deformability. However, the data on the changes in RBC membrane fluidity upon adenylyl cyclase signaling is absent. The aim of the current study was to assess the effects of stimulation of different pathways of adenylyl cyclase cascade on the diffusion of lipids of erythrocytes cytoplasmic membrane. The obtained results were compared with the changes in the fluidity of membrane of erythrocytes after fixation using glutaraldehyde.

## Methods

### Erythrocytes sampling and preparation

RBC were extracted from the blood, sampled from a healthy male donor (age 25) by finger prick method using a sterile lancet. After the finger pricking, 10 μl of the donors' blood was collected into 1 ml of isotonic PBS (phosphate-buffered saline, pH 7.4, Gibco) to obtain 1% suspension and washed in PBS (three times, 3 min, 3000 x g, room temperature).

After washing, the RBC were labeled using a non-specific lipophilic fluorescent dye CM-DiI (Vybrant 22,888, Invitrogen). The stock solution of the dye was prepared in dimethyl sulfoxide (DMSO). The labeling solution was prepared by subsequent dilution of the stock solution with PBS to achieve the final CM-DiI concentration 1 μM. DMSO concentration in the labeling solution was < 0.5% to avoid possible cytotoxicity. For labeling, the washed RBC were put into CM-DiI solution preliminary warmed to 37 °C in ratio 1:100, and incubated for 30 min at 37 °C according to the provided recommendations (Andrade et al. 1996). After labeling, the RBC were washed in PBS (three times, 3 min, 3000 x g, room temperature) to get rid of the unbound dye molecules.

### Composition of the experimental samples

The labeled RBC in volume of 10 μl were added to the experimental PBS solutions (1 ml) of three different adenylyl cyclase cascade stimulation agents: dibutyryl-cAMP (db-cAMP, membrane-permeable analog of cAMP, D0627, Sigma Aldrich) 300 μM concentration; epinephrine (non-selective adrenoreceptor agonist, E4250, Sigma Aldrich) 25 μM; metaproterenol (selective β2-adrenoreceptor agonist, M2398, Sigma Aldrich) 50 μM. The RBC were incubated in the aliquots of the studied substances during 15 min at 37 °C. The control sample represented the intact labeled RBC in PBS. After incubation, the experimental suspension was put between two glass slides for further microscopic fluorescent measurements.

Fixation of the labeled RBC in glutaraldehyde was used as a negative control to compare with effects of adenylyl cyclase cascade activation on the membrane properties. Labeled RBC were inserted into 0.7% solution of glutaraldehyde and incubated for 15 min at 37 °C. After incubation, the RBC were gently sedimented and added to PBS to achieve a 1% suspension for further measurements.

### FRAP measurements and data analysis

Fluorescence recovery after photobleaching (FRAP) measurements were performed using microscope Eclipse Ti-E with confocal module A1 (Nikon Corporation, Tokyo, Japan) at 20 °C. Bleaching was controlled using proprietary Nikon software and performed at 488 nm with 1 s duration. Fluorescent imaging was performed with excitation at 488 nm and detection at 543 nm long pass. The shape of the bleaching area, as well as the regions of interests (ROI) of fluorescence measurements were the same for every cell in each series of the experiments. For a control of non-reversible bleaching the image of a fully bleached RBC is provided in Supplementary materials (Fig. S1).

FRAP curves were extracted from raw images and normalized using ImageJ scripts. The obtained curves were processed manually in Origin software (Origin Lab) for obtaining the characteristic recovery time and mobile fraction values. To do that, the initial curves were inverted and approximated using the multiexponential decay function:

1 $$\tilde{I}\left( t \right) = \sum {A_i e^{ - {\raise0.7ex\hbox{$t$} \!\mathord{\left/ {\vphantom {t {\tau_i }}}\right.\kern-0pt}\!\lower0.7ex\hbox{${\tau_i }$}}} } ,$$

where $\widetilde{I}\left(t\right)=1-I(t)$; $I(t)$—the normalized FRAP curve. The multiexponential approach was used in the analysis of fluorescence recovery kinetics in order to consider the asymmetric lateral mobility of erythrocytes membrane lipids (Morrot et al. 1986). The fitting quality was controlled by the minimum value of reduced *χ*^2^ and coefficient of determination (COD), which was controlled by the proximity of *R*^2^ to 1. The recovery time ${T}_{1/2}$ was evaluated as an amplitude-weighted relaxation time of a corresponding kinetic:

2 $${T}_{1/2}=\frac{\sum {A}_{i}{\tau }_{i}}{\sum {A}_{i}}.$$

The mobile fraction ${M}_{{\text{f}}}$ was calculated using the formula (Ishikawa-Ankerhold et al. 2012):

3 $${M}_{f}=\frac{{I}_{\infty }-{I}_{0}}{{I}_{i}-{I}_{0}},$$

where ${I}_{i}$—initial pre-bleach intensity value; ${I}_{0}$—post-bleach intensity value; ${I}_{\infty }$—maximal plateau intensity value in the FRAP curve.

The values of the apparent diffusion constants ($D$) of membrane lipids were obtained by automated algorithmic processing of FRAP recordings using FRAPbot software described in (Kohze et al. 2017) and validated manually using approaches described in (Kang et al. 2012). At least 10 different RBC were analyzed for each experimental sample. FRAP measurements were performed only on RBC of approximately same size with normal biconcave shape to partially consider the impact of erythrocyte age since RBC decrease their volume during aging/storage due to dehydration, release of metabolites and vesiculation (Huang et al. 2011).

## Results

**Fig. 1:**
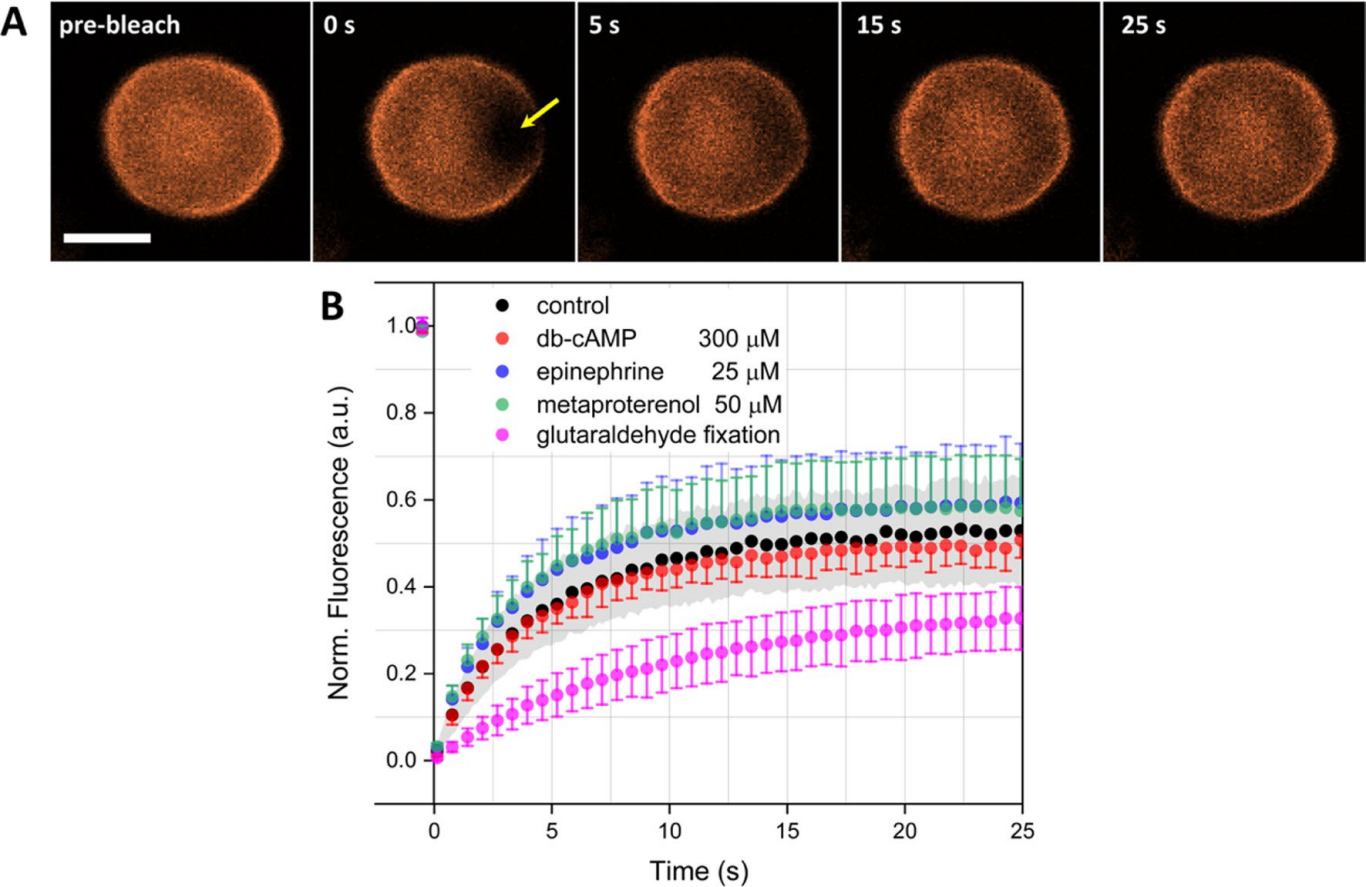
**A** Series of images demonstrating a typical FRAP measurement performed on a single RBC, stained with the lipophilic dye CM-DiI, at 20℃. Yellow arrow indicates the photobleached area. Scale bar 3 μm. **B** Mean FRAP curves, measured on no less than *N* = 10 different cells each. Gray-colored background corresponds to the standard deviations of control sample measurements results, color error whiskers correspond to standard deviations of experimental samples data

Figure 1A demonstrates a series of images showing a typical FRAP measurement on a single RBC: CM-DiI stained RBC before photobleaching (pre-bleach); at the time of photobleaching (0 s) with yellow arrow indicating the photobleached area; different stages of postphotobleaching recovery (5, 15 and 25 s). Figure 1B demonstrates mean FRAP curves, measured on different RBC in control (black dots) and experimental (color dots) samples. Curves were measured on no less than 10 cells in each sample group. Dots represent mean values; error whiskers correspond to standard deviations. For clearer representation of the data, error of the control sample is visualized as gray background, and for some experimental samples, the errors whiskers are visualized only as plus or only minus not to overload the diagram.

**Fig. 2:**
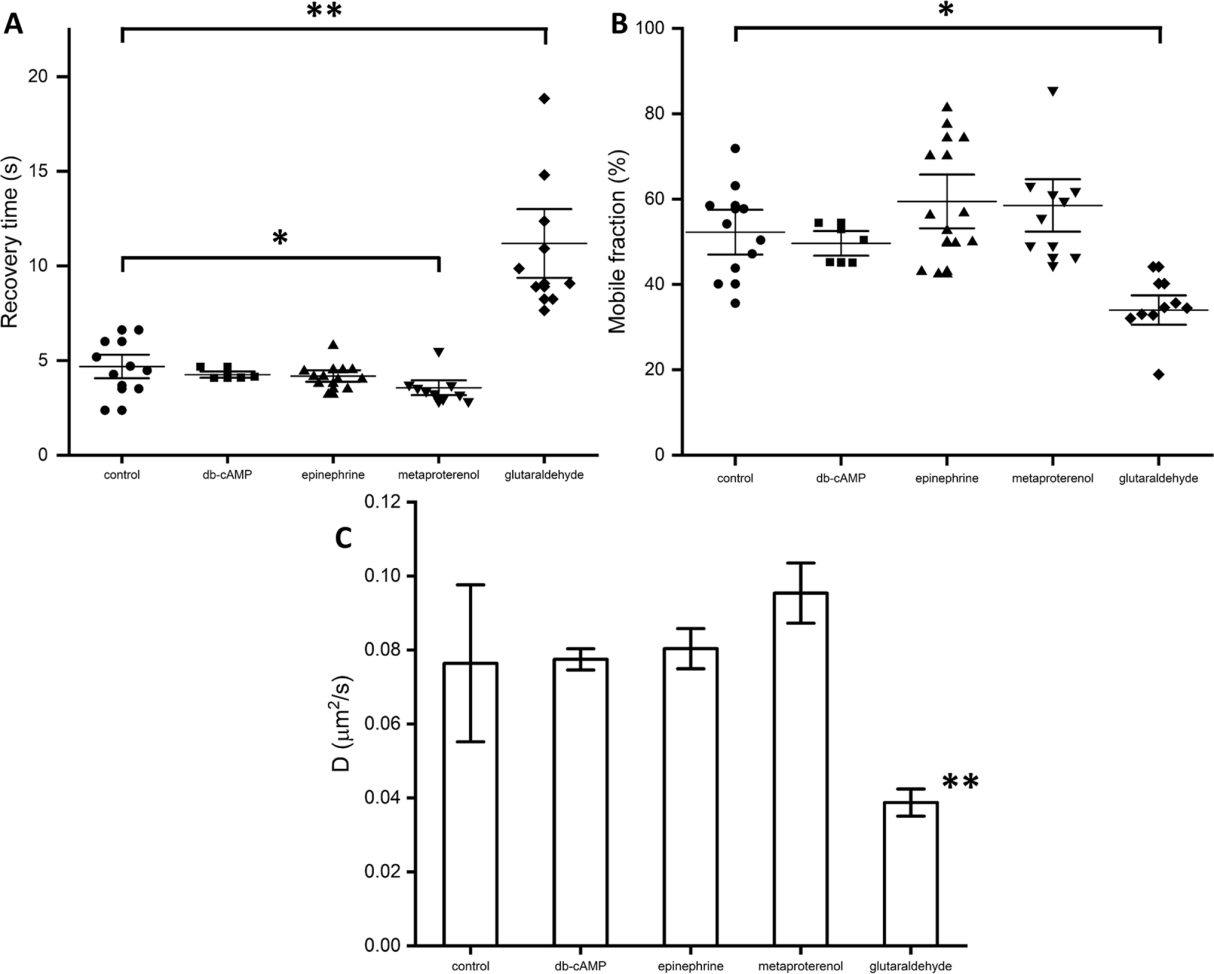
Results of FRAP data analysis of the effects of stimulation of different pathways of adenylyl cyclase signaling cascade in RBC. **A** Fluorescence recovery time values. **B** Mobile fraction ratio values. Each point corresponds to a single cell measurement, horizontal lines correspond to median values, whiskers correspond to standard errors. **C** Lateral diffusion coefficient of membrane lipids (20 °C). Data is represented as column diagram indicating mean values, error bars correspond to standard deviations. *p < 0.04; **p < 0.004 (standard T-test for dependent variables)

Incubation of RBC in the solutions of adenylyl cyclase cascade stimulators changed the profile of FRAP postphotobleaching recovery (Fig. 1B): the recovery process was accelerated in comparison with the control RBC. The most pronounced effect was achieved under the adrenergic receptor stimulation with epinephrine (blue dots) and metaproterenol (green dots), and the effect of metaproterenol was more distinct. The acceleration effect of db-cAMP (red dots) was less significant and took place only during the early phase (first 10 s) of the recovery. Meanwhile, the effect of RBC fixation in glutaraldehyde (magenta dots) was expressed dramatically: the FRAP recovery was slowed down during the whole period of the observation and required much more time to reach the plateau.

Figure 2 demonstrates the results of the analysis of the FRAP data. First, one can see a clear trend in a decrease in the recovery time ${T}_{1/2}$ of fluorescent signal upon the effect of adenylyl cyclase cascade activation (Fig. 2A): in the control sample, the recovery time was 4.7 ± 0.4 s and decreased after the adenylyl cyclase cascade was stimulated. The fastest recovery was observed upon the effect of [metaproterenol] 50 μM (3.5 ± 0.2 s), and the change was statistically significant (*p* < 0.04, standard T-test for dependent variables). In case of other stimulation agents, the recovery acceleration effect was less significant yet observable: 4.2 ± 0.2 s (epinephrine) 25 μM; 4.3 ± 0.1 s (db-cAMP) 300 μM. The negative control test demonstrated that the fixation of RBC in 0.7%-glutaraldehyde solution deaccelerated fluorescence recovery drastically: the recovery time significantly (*p* < 0.004) increased to 11.2 ± 1.2 s. Meanwhile, different stimulation of adenylyl cyclase cascade did not change the mobile fraction significantly which was approx. 50–60% (Fig. 2B). Significant (*p* < 0.04) decrease of mobile fraction to 30% was observed only upon glutaraldehyde fixation (Fig. 2B). The obtained results totally correspond with the behavior of lipids lateral diffusion coefficient ($D$) values (Fig. 2C). Analysis of FRAP data using FRAPbot software revealed, that in the control samples $D=$ 0.076 ± 0.014 μm^2^/s and increased when the adenylyl cyclase cascade was activated: 0.077 ± 0.002 μm^2^/s (db-cAMP); 0.080 ± 0.004 μm^2^/s (epinephrine); 0.095 ± 0.005 μm^2^/s (metaproterenol) although the effect was not statistically significant at current concentrations. Fixation of RBC in glutaraldehyde solution led to a significant (*p* < 0.004) almost twice decrease in lipids diffusion (0.039 ± 0.002 μm^2^/s).

## Discussion

Adenylyl cyclase signaling cascade is considered as an essential metabolic system to establish regulation of RBC deformability (Cilek et al. 2023). This cascade is associated with G-protein coupled receptors (GPCR), including the adrenergic receptors family triggered by the recognition of the catecholamines such as epinephrine (adrenaline) (Horga et al. 2000) or other adrenergic agonists (metaproterenol, isoproterenol) (Horga et al. 2000; Muravyov and Tikhomirova 2012). Interaction between the receptor and its ligand activates the membrane enzyme adenylyl cyclase (AC), which converts ATP into the form of cyclic adenosine monophosphate (cAMP). The latter acts as a secondary messenger and activates cAMP-dependent protein kinase A (PKA), which phosphorylates the residues of proteins, namely β‐spectrin, adducin, protein 4.1, and dematin proteins, responsible for the attachment of cells cytoskeleton to the membrane. As a result, the corresponding protein complexes disintegrate and the interaction between cytoskeleton and membrane becomes weaker resulting in an increase in RBC deformability.

Several studies suggest that phosphorylation of the 4.1 band protein by cAMP-dependent kinase can be a central regulatory link in the re-arrangements of the erythrocyte cytoskeleton (Boivin 1988; Ling et al. 1988). Additional evidence is provided in (Muravyov et al. 2010) on the increase in RBC deformability under the effects of dibutyryl-cAMP, membrane-permeable analog of cAMP. Stimulation of AC cascade resulted in the increase in filterability of animal and human RBC (Ambrus et al. 1990; Oonishi et al. 1997). Modulation of the AC-cAMP-PKA pathway was potentialized for improvements of impaired deformability of RBC of blood of the patients suffering from sickle cell disease (Goksel et al. 2023; Ugurel et al. 2019). In a recent study conducted by analyzing the proteomic assay of RBC, it was concluded that the AC-cAMP-PKA metabolic pathway is associated with the changes of the phosphorylation status of membrane proteins as a response to the shear-induced mechanical stress, embodying mild phosphorylation of serine and tyrosine residues in the RBC proteome leading to the improvements of RBC deformability under physiological shear stress (Ugurel et al. 2022).

Meanwhile, the mechanisms of the changes of viscous properties of RBC membrane under the effects of the AC-cAMP-PKA pathway are not understood completely. In the work (Hirata et al. 1979), it was demonstrated that the interaction of adrenergic receptor with its agonist stimulated the phospholipid methylation in the membrane of rat reticulocyte ghosts, resulting in the formation of a lipid modification *phosphatidyl-N-monomethylethanolamine*, which markedly decreased membrane microviscosity. In the work (Tuvia et al. 1999), stimulation of the AC cascade by exposure of RBC to epinephrine resulted in significant increase in fluctuations of the membrane. In (Hilário et al. 2003), it was shown that adrenaline increased erythrocyte membrane fluidity in samples of blood of healthy male donors. The effects of adrenergic receptors stimulation are in the agreement with the influence of cAMP-mediated protein phosphorylation since it was demonstrated (Koshino et al. 2012) that activation of PKA clearly decreased RBC membrane mechanical stability.

**Fig. 3:**
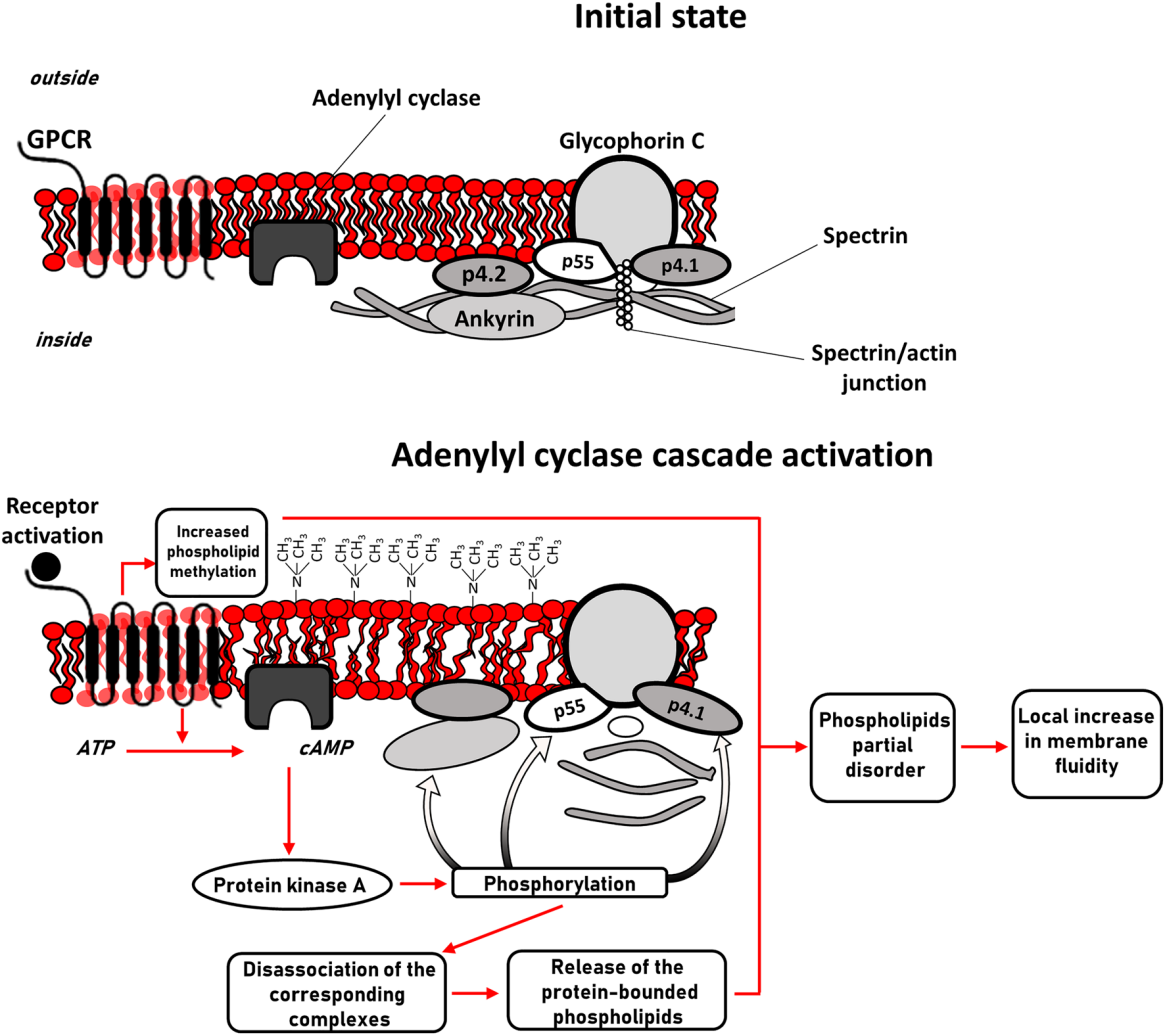
Scheme of the mechanisms of influence of adenylyl cyclase cascade activation on the fluidity of RBC membrane. Initial state (before cascade activation) is characterized by strong interaction between cytoskeleton and intermembrane anchoring proteins: the whole construction is tense, and the phospholipids are primarily ordered. Adenylyl cyclase cascade activation leads to the weakening of interaction between the cytoskeleton and membrane proteins, which in addition to increased methylation results in the local disordering of phospholipids with a subsequent increase in membrane fluidity

Our results presented in the current study are in the agreement with the common concept of AC functioning in RBC. Figure 3 provides a scheme of reactions leading to the local increase in membrane fluidity upon adenylyl cyclase cascade activation. The initial state, when the cascade is not activated, is characterized with strong interaction between RBC cytoskeleton and intermembrane anchoring proteins when phospholipids are ordered with consequently high local microviscosity. Upon cascade activation, the phosphorylation-driven disassociation of cytoskeleton protein complexes leads to the local release of the corresponding protein-bounded phospholipids because the interaction between intermembrane proteins and the tense cytoskeleton becomes weaker. The final step is characterized by a local increase in membrane fluidity followed by the partial disordering of the phospholipids.

The opposing effect of the fixation of RBC in glutaraldehyde was clearly observed as the membrane fluidity decreased significantly. The clear deacceleration of the fluorescence recovery indicates the significant rigidity of the membrane caused by glutaraldehyde fixation. These results fully correspond with a significant amount of data on the effect of glutaraldehyde on RBC mechanics. Along with the fixation comes an increase in the stiffness of the cells, which in the context of red blood cells is expressed as an increased rigidity (Abay et al. 2019) with subsequent decrease in shear-induced deformability (Maslianitsyna et al. 2020; Sosa et al. 2014). Glutaraldehyde can also interact with particular lipid macromolecules, which contain free amino groups, however, generally lipids immobilization is implemented primarily by the creation of crosslinked protein complexes that either capture proteins bound to lipids or otherwise slow or prevent their diffusion (McKenzie 2019). Thus, the significant decrease in membrane fluidity under glutaraldehyde was caused mostly by the decreased mobility of the protein complexes embedded in the membrane. FRAP technique allows to assess the changes in the membrane fluidity induced by adenylyl cyclase cascade stimulation. However, the impact of different pathways upon the cascade stimulation was different. Adrenergic receptors activation provided the strongest stimuli, while the stimulation via membrane-permeable analog of cAMP (db-cAMP) was pronounced less significantly. We assume the reason is that the mechanism of db-cAMP action involves stimulation of cAMP-dependent protein kinase A in the cytosol. Another option is the reversible character of the stimulation of cAMP-dependent PKA (Humphries et al. 2007). The latter corresponds with our results on the time-dependence of db-cAMP effect on kinetics of the fluorescence recovery (Supplementary material, Fig. S2): the most significant effect was observed during the first hour of the measurements, while later on it started to decrease. In this regard, further clarifying of the changes of RBC membrane properties upon adenylyl cyclase cascade stimulation requires studies involving different concentrations of the agents, different incubation and measurement times and different cAMP analogs.

## Conclusion

In the course of evolution, in order to achieve the maximum efficiency of the respiratory gasses transport, mammalian erythrocytes have lost many cell organelles to provide the volume for filling with hemoglobin. Nevertheless, erythrocytes retained molecular signaling cascades, which operation ensures the ability to regulate their deformability in accordance with the metabolic demands of the organism to maintain microcirculation and provide optimal rheological conditions for the blood flow. Adenylyl cyclase cascade plays a major role in the regulation of erythrocyte biomechanics. Its activation leads to the significant re-organization of cytoskeleton proteins configuration, accompanied with local disordering of the phospholipids. The latter results in an increase in the membrane fluidity. This aspect of adenylyl cyclase cascade functioning should be considered when developing novel approaches for the correction of blood microrheology impairments.

### Supplementary Information

Below is the link to the electronic supplementary material.

Supplementary file1 (PDF 998 KB)

## Data Availability

All the necessary data are provided in the figures in the present paper. Generative AI and/or AI-assisted technologies were not used in the writing process.
