# Supplementary material for: Erythrocytes membrane fluidity changes induced by adenylyl cyclase cascade activation: study using fluorescence recovery after photobleaching
Source: Eur Biophys J. 2024 Apr 16;53(4):239–47. doi: 10.1007/s00249-024-01707-x (PMC11098875; doi:10.1007/s00249-024-01707-x)
Supplement: Supplementary file 1 — Supplementary file1 (PDF 998 KB) [file 249_2024_1707_MOESM1_ESM.pdf]

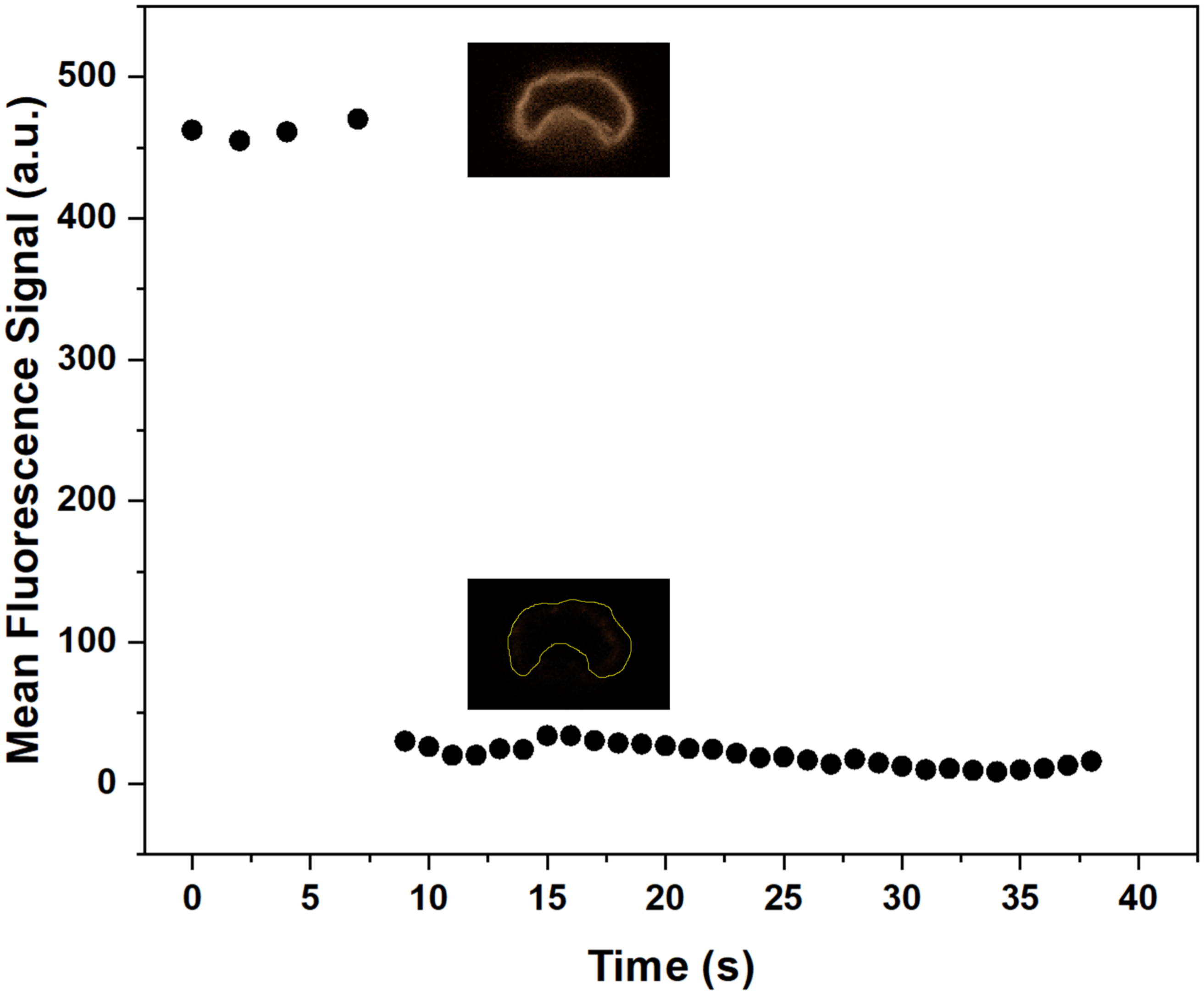

Fig. S1. The control for irreversible photobleaching (the capturing of the fully bleached RBC). Upper inlet demonstrates the image of the cell before the bleaching (pre-bleach). The whole cell was bleached, and the yellow line in the lower inlet figure describes the ROI corresponding to the area of a bleached cell. Fluorescence signal was assessed as a mean pixel value in the ROI.

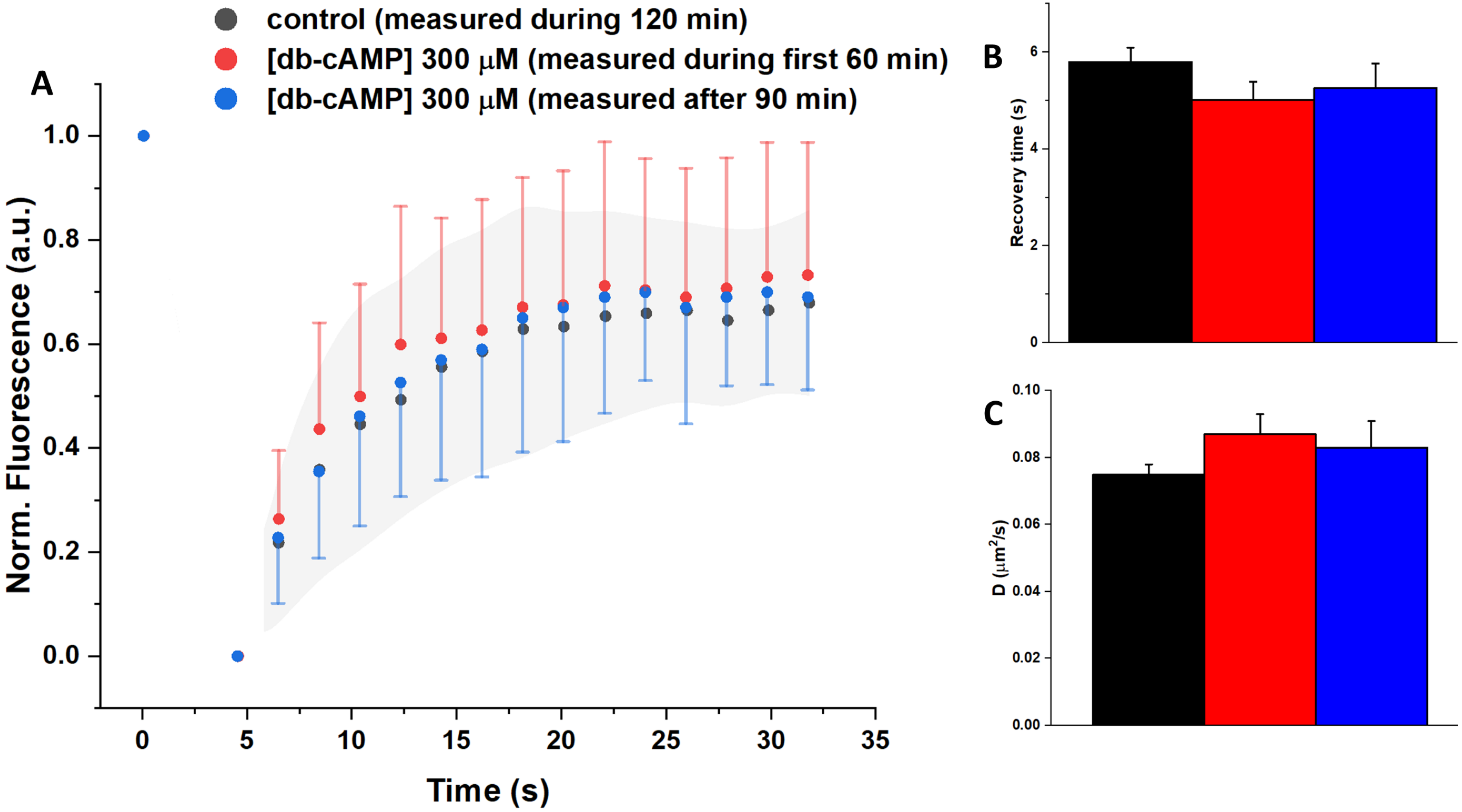

Fig. S2. Changes in the fluidity of membrane of RBC, exposed to db-cAMP, assessed during different measurement time periods. A - FRAP kinetics; B – Recovery time values; C – Lateral diffusion coefficients. Measurements were performed on N=10 erythrocytes of the same size and shape. The graphs demonstrate mean values, error bars correspond to SD. Control measurements errors are visualized as grey background (also corresponds to SD). Measurements performed at 20 ^{o}C.
